# Enhancing the destruction of *Burkholderia cepacia* biofilm on stainless steel coupons by combining matrix-degrading enzymes with antimicrobials

**DOI:** 10.3389/fcimb.2025.1662291

**Published:** 2025-11-10

**Authors:** Yukta P. Gharat, Ahmed G. Abdelhamid, Ahmed E. Yousef

**Affiliations:** 1 Department of Food Science and Technology, The Ohio State University, Columbus, OH, United States; 2 Department of Food Science and Human Nutrition, Michigan State University, East Lansing, MI, United States; 3 Department of Microbiology, The Ohio State University, Columbus, OH, United States

**Keywords:** *Burkholderia cepacia*, biofilm, food spoilage, antimicrobial, synergism, matrix-degrading enzymes

## Abstract

*Burkholderia cepacia* is an underexplored opportunistic pathogen and a food spoilage species. The bacterium may serve as an ideal model for biofilm formation and resilience. Herein, we explored the possibility of enhancing the destruction of preformed *B. cepacia* biofilm by combining enzymes (amylase, DNase, and protease) that potentially degrade biofilm matrices with diverse antimicrobials. Initially, the biofilm-forming ability of *B. cepacia* ATCC 25416 was assessed in two microbiological media. A nutrient-rich broth favored planktonic cell proliferation, whereas a nutrient-limited medium supported robust biofilm formation. The minimum inhibitory concentration (MIC) of the tested antimicrobials against planktonic cells (MIC-Plank) was determined. Ciprofloxacin and meropenem gave the smallest MIC-Plank of 4.0 and 8.0 μg/mL, respectively. The MIC of the two antimicrobials, when applied against preformed biofilm (MIC-Bio), increased to 16 μg/mL. Enzyme-antimicrobial combinations decreased the MIC-Bio of the antimicrobials to 4.0–8.0 μg/mL in a synergistic or additive manner, as measured by the fractional inhibitory concentration index (FICI). Among the tested combinations, α-amylase-ciprofloxacin exhibited a synergistic effect (FICI = 0.50), proteinase K-ciprofloxacin (FICI = 0.625), and α-amylase-meropenem (FICI = 0.750) showed an additive effect against *B. cepacia* biofilms. These combinations, at their MIC-Bio, were applied to preformed biofilms on stainless-steel coupons. Application of α-amylase, ciprofloxacin, and their combination significantly decreased (*p* < 0.0001) the biofilm populations from 8.4 ± 0.2 (untreated coupons) to 6.03 ± 0.2, 5.3 ± 0.3, and 4.5 ± 0.4 log_10_ CFU/coupon, respectively. Similarly, α-amylase, meropenem, and their combination significantly decreased (*p* < 0.0001) the biofilm populations from 7.5 ± 0.5 (untreated coupons) to 5.8 ± 0.1, 5.6 ± 0.1, and 3.8 ± 1.0 log_10_ CFU/coupon, respectively. These findings were confirmed when biofilms formed on stainless-steel coupons were examined through scanning electron microscopy. It is predicted that antimicrobial concentrations higher than MIC-Bio in the treatment combinations would eliminate residual biofilm on the coupons, but this needs to be studied. To conclude, enzyme-antimicrobial combinations offer a promising biofilm control strategy by mitigating *B. cepacia* preformed biofilm and minimizing risks associated with this potentially hazardous and spoilage bacterium. Such a strategy could be implemented in processing environments when food-grade antimicrobial additives are used instead of the currently tested antimicrobials.

## 1 Introduction

Microbial contamination originating from biofilms has become a challenge in all sectors of the food industry, including fresh produce (Srey et al., 2013), seafood (Shikongo-Nambabi et al., 2010), dairy (Chmielewski and Frank, 2003), poultry (Harvey et al., 2007), and meat (Sofos and Geornaras, 2010) industries. While extensive research addressed well-characterized biofilm-forming pathogens, emerging foodborne microorganisms such as *Burkholderia* spp., remain largely understudied. Given their increasing significance in healthcare and food safety (Moore et al., 2001), detailed investigations are needed to understand their role in biofilm formation and persistence in food-related environments.

The genus *Burkholderia* is composed of over 30 species that live in remarkably diverse ecological niches ranging from contaminated soils to the human respiratory tract (Coenye and Vandamme, 2003). *B. cepacia*, an environmental soil bacterium commonly found in plant rhizospheres, is responsible for “slippery skin” rot in onions and soft rot in various vegetables (Jacobs et al., 2008). Initially classified as *Pseudomonas cepacia*, the species was reclassified in 1992 after phenotypic and genotypic studies demonstrated its distinct taxonomic placement (Yabuuchi et al., 1992). Beyond its role in plant disease, *B. cepacia* has emerged as a clinically significant opportunistic pathogen, particularly in cystic fibrosis (CF) patients and immunocompromised individuals, where its intrinsic antimicrobial resistance complicates treatment (Isles et al., 1984). Despite these concerns, the role of the bacterium as a biofilm-forming contaminant in food processing environments remains underexplored.

Biofilm bacteria are encased in a self-produced extracellular polymeric substance (EPS), which enhances microbial survival by shielding cells from antimicrobial agents and the host’s immune response (Flemming et al., 2007). Additionally, biofilm bacteria exhibit remarkable adaptability to their surrounding environments. This resilience allows them to adapt to environmental changes and to survive in harsh conditions (Stoodley et al., 2002). In recent years, researchers have explored many physical, chemical, and biological strategies to tackle bacterial biofilms. Sanitizers and disinfectants such as quaternary ammonium compounds, chlorine-based agents, peracetic acid, and hydrogen peroxide are commonly used in food processing facilities for biofilm control because of their strong antimicrobial activity and effectiveness against a wide range of bacteria. However, prolonged or improper use of these chemicals has contributed to bacterial resistance and raised the risks to human health and environment (Dawan et al., 2025). Emerging surface decontamination technologies for the eradication of biofilm bacteria include pulsed ultraviolet light, electron beam, steam heating, irradiation at 405 nm, and treatment with ozone, ultrasound, or gaseous chlorine dioxide (Liu et al., 2023). Other researchers reported the efficacy of bacteriophages and phage lysozymes as antibiofilm agents (Yin et al., 2021). Matrix-degrading enzymes have been employed to disrupt biofilms; however, their efficiency depends on the composition of the EPS (Banar et al., 2016). For each polymeric component in EPS, there are enzymes that assist in its breakdown; these include proteases, which hydrolyze bacterial proteins (Solanki et al., 2021), lysozymes, which degrade the cell envelope’s peptidoglycan (Khorshidian et al., 2022), and alginate lyases and amylases that degrade polysaccharides (Lahiri et al., 2021).

In a previous study (Iñiguez-Moreno et al., 2021), the effectiveness of removing mixed-species biofilms was assessed using a combination of alkaline protease and α-amylase. The combination treatment removed 93.4% to 96.3% of biofilm population on stainless-steel. Enzymes like protease and DNase disrupted biofilm EPS; these can be applied individually or included in sanitization strategies to enhance the inactivation of microbial cells within biofilms (Kim and Kim, 2022). A glycosyl hydrolase, originally derived from a *Salmonella* phage-encoded enzyme, was shown to effectively inhibit biofilm formation and disrupt mature biofilms of *Escherichia coli* O157:H7, *Listeria monocytogenes*, and *Salmonella enterica* serovar Typhimurium (Mayton et al., 2021). To be effective, combined treatments need to be tailored against each biofilm former and its EPS.

Despite many advancements in biofilm control, *B. cepacia* remains a poorly characterized biofilm producer, raising concerns about potential contamination of food during persistence of this spoilage and opportunistic pathogen in food processing environments. Understanding the factors that influence *B. cepacia* biofilm development can provide critical insights into biofilm’s resilience and contribution to antimicrobial resistance. The current study was initiated to address these knowledge gaps. Hence, we examined the impact of various parameters, including media composition, inoculum sizes, and shaking during incubation on biofilm formation, using both polystyrene 96-well plates and stainless-steel coupons. Furthermore, we evaluated synergistic antimicrobial strategies by combining the biofilm-degrading enzymes, α-amylase, DNase I, and Proteinase K with selected commercial antimicrobial agents. The ability of these combinations to degrade preformed *B. cepacia* biofilms synergistically was assessed as an approach to mitigate the risks posed by biofilm-forming spoilage or pathogenic bacteria in food processing environments.

## 2 Material and methods

### 2.1 Bacterial strain

*B. cepacia* ATCC 25416 was sourced from the culture collection at the Food Microbiology Laboratory of The Ohio State University (Columbus, OH, USA). The bacterium is linked to the spoilage of onion bulbs and unpasteurized raw milk (Moore et al., 2001), and it is recognized for its robust ability to form biofilms (Tavares et al., 2020). The strain was streaked from frozen stock stored at –80 °C in 25% glycerol (Sigma-Aldrich, Burlington, MA, USA) onto a Tryptic Soy Agar (TSA) (Bacton, Dickson and Company, Franklin Lakes, NJ, USA) followed by aerobic incubation in static condition at 37 °C for 24 hours. Fresh frozen stock of the culture was used to initiate each experiment.

### 2.2 Growth media and incubation conditions

*B. cepacia* ATCC 25416 was grown using two media (**Table 1**); (i) tryptone yeast extract dextrose (TYD) broth, a nutrient rich medium, which was derived from Ashdown’s medium (Howard and Inglis, 2003) and *B. cepacia* selective agar medium (Thermo-Fisher Scientific, Waltham, MA, USA) after excluding the selective agents and modifying the composition, and (ii) yeast extract dextrose calcium carbonate broth (da Silva et al., 2021), a nutrient limited medium that was modified (mYDC broth) by excluding calcium carbonate, which tended to precipitate after autoclaving. Agar versions of these broths were made by including agar at a 2.0% level.

**Table 1:** Composition of media^a^ used in the current study for growth and biofilm formation by *Burkholderia cepacia*.

Ingredient	Amount, g/L	
	Tryptone yeast extract dextrose (TYD) broth	Modified yeast extract dextrose calcium carbonate (mYDC) broth^b^
Tryptone	5	–
Protease peptone	3	–
Yeast extract	7	10
Dextrose	7	20
Fructose	3	–
Glycerol	1	–
Sodium chloride	5	–
K_2_HPO_4_	2.5	–
pH	7.0	7.0

^a^Media and ingredients were procured from Bacton, Dickson and Company, Franklin Lakes, NJ, USA; Sigma-Aldrich, Burlington, MA, USA; and Thermo Fisher Scientific, Waltham, MA, USA.

^b^reported previously by da Silva et al., 2021, but modified in the current study by excluding calcium carbonate.

The growth curves of *B. cepacia* ATCC 25416 were determined in TYD and mYDC broth media as follows. To prepare the inoculum, the bacterium was grown in mYDC broth under aerobic conditions for 24 hours at 37 °C with shaking at 180 rpm. The overnight culture was diluted in TYD or mYDC broth to a final population of 10^3^–10^4^ CFU/mL. Aliquots (200 μL) of the diluted culture were dispensed in the well of a polystyrene 96-well plate and incubated statically at 37 °C. Samples (100 μL, each) were taken (from separate wells) after incubation for 0, 3, 6, 9, 12, 15, 20, 24, 36, and 48 hours. These samples were ten-fold serially diluted and plated on TYD and mYDC agar. The plates were incubated at 37 °C for 24 hours, and *B. cepacia* populations were counted. The population counts over time (growth curves), derived from three independent replicates, were fitted using the following Gompertz model (Ali et al., 2025):


$$Y=a+\left(b-a\right)e^{-e-c\;(t-d)}$$

where the dependant variable “*Y”* is *B. cepacia* population (log_10_ CFU/mL), the independent variable “*t”* is time (hour), and model’s parameters were “*a”* is the lower asymptote (log_10_ CFU/mL), “*b”* is the upper asymptote (log_10_ CFU/mL), “*c”* is the growth rate (log_10_ CFU/mL/hour), and “*d”* is the inflection point (hour). The model’s mathematical parameters (*a*, *b*, *c*, and *d*) were determined using statistical software (JMP Pro 17; SAS Institute Inc., Cary, NC).

### 2.3 Biofilm formation: optimization of bacterial inoculum and incubation conditions

Biofilm optimization in the two microbiological media (**Table 1**) was conducted using polystyrene 96-well microtiter plates (Corning Costar; Fisher Scientific). The colorimetric quantification method, using crystal violet, was used to assess biofilm formation (Rose et al., 2009). This experiment was designed as described previously (Reddersen et al., 2021) with modifications. The following four experimental variables were evaluated: inoculum size (10^4^–10^7^ CFU/mL), aerobic incubation conditions (static vs. shaking), incubation time (24 vs. 48 hours), and growth media (TYD vs. mYDC) for their impact on biofilm. The 24-h-old *B. cepacia* culture was adjusted to 0.1 OD_600nm_ (10^7^ CFU/mL as confirmed by plating on mYDC agar). Serial dilutions were performed to obtain inoculum sizes of 10^6^, 10^5^, and 10^4^ CFU/mL. A 200-μL aliquot of each diluted culture in each microbiological medium was added to the wells of polystyrene 96-well microtiter plates. Plates were incubated under aerobic static conditions at 37°C or under shaking conditions (140 rpm) at 37°C for 24 or 48 hours to allow biofilm formation. Sterile uninoculated media served as a negative control. Following the incubation, planktonic cells were carefully aspirated from each well, and wells were washed three times with sterile saline solution (0.85%) to remove non-adherent cells. Plates were then allowed to dry at 55°C for 20 min. Biofilms were stained by adding 250 µL of 0.1% crystal violet solution (Becton, Dickinson and Company, Franklin Lakes, NJ, USA) to each well, followed by incubation at room temperature (25 ± 2°C) for 25 min. The plate wells were washed with sterile water to remove any unbound or excess stain, and then the excess water was carefully removed. Subsequently, crystal violet in each well was solubilized in 300 µL of 70% ethanol (Decon Laboratories Inc., King of Prussia, PA, USA) for 45 min. Biofilm biomass was quantified by measuring absorbance at OD_590nm_ using a microtiter plate reader (SpectraMax 384 Plus; Molecular Devices, San Jose, CA, USA). Each condition was tested in four replicates.

### 2.4 Determining minimum inhibitory concentration of antimicrobials against planktonic cells (MIC-Plank) using resazurin dye

The antimicrobials used were tetracycline hydrochloride (Sigma-Aldrich, Inc.), chloramphenicol (IBI Scientific, Peosta, IA), kanamycin sulfate (Sigma-Aldrich, Inc.), ceftazidime (European pharmacopoeia reference standard), erythromycin (Sigma-Aldrich, Inc.), ampicillin (Sigma-Aldrich, Inc.), ciprofloxacin hydrochloride (MP biochemicals, USA), and meropenem trihydrate (ThermoFisher Scientific). Each antimicrobial was prepared by dissolving in the appropriate solvent or water to obtain stock concentrations of 2000 μg/mL (Jorgensen, 2012). Resazurin solution was prepared by dissolving 0.05 g resazurin powder (Sigma Aldrich, Inc.) in 50 mL sterile distilled water. The solution was thoroughly mixed and then diluted 1:10 to obtain a final working concentration of 0.01% for further experiments. To protect the dye degradation from light exposure, the resazurin solution was stored in a sterile tube wrapped with aluminum foil.

The resazurin-based microdilution assay was used to evaluate the inhibitory effects of the antimicrobials against *B. cepacia* planktonic cells using the broth microdilution method (Elshikh et al., 2016). The *B. cepacia* strain was grown in the mYDC broth for 24 hours at 37°C with shaking at 180 rpm. Following incubation, the inoculum was prepared by diluting the overnight culture 1:1000 in the mYDC to achieve a cell density of ~10^4^ CFU/mL. The selected antimicrobials were serially diluted two-fold in mYDC broth to generate final concentrations ranging from 0.24–1000 μg/mL. In the polystyrene 96-well plates, 100 µL of the appropriate antimicrobial dilution and 10 µL of the diluted culture were added. The plates were briefly shaken to ensure uniform mixing before incubation at 37°C for 24 hours. After incubation, turbidity readings were taken at OD_600_ nm and then 10 μL of resazurin dye (0.01%) were added to each well and incubated at room temperature for 30 min. Active bacterial cells will reduce the non-fluorescent resazurin dye (blue) to the fluorescent resorufin (pink) (O’Brien et al., 2000). MIC values were interpreted using the standard CLSI M100 Performance Standards for Antimicrobial Susceptibility Testing, 33^rd^ edition, 2023 (Lewis et al., 2023). The lowest concentration before turbidity and resazurin color change was considered as the MIC. The color change of the dye was assessed visually, with a shift from blue to pink, indicating cell growth and no change indicating absence of growth (Chakansin et al., 2022). Microdilution was performed in triplicate for each antimicrobial.

### 2.5 Enzyme-antimicrobial synergy against preformed biofilms using checkerboard assay

The enzymes α-amylase from *Bacillus* spp. (Sigma-Aldrich, Inc), proteinase K (Invitrogen, Carlsbad, CA), and DNase I (Thermo Fisher Scientific) were included in this test. The enzymes were solubilized according to the manufacturer’s instructions, as follows. The α-amylase (10 mg/mL) was prepared by solubilizing the powder in a buffer solution consisting of 23 mM potassium phosphate and 6.6 mM NaCl, and the pH was adjusted to 6.9. To prepare DNase-I, 100 μL of the commercial enzyme preparation was diluted in 900 μL of DNase reaction buffer (Thermo Fisher Scientific), and further diluted 1:10 to achieve a final concentration of 0.01 U/μL. Proteinase K was prepared by dissolving the commercial preparation in 50 mM Tris-HCl (pH 8.0), containing 2 mM calcium acetate (Mallinckrodt chemicals, St. Louis, MO, USA), to prepare a solution containing 2 mg/mL. All enzyme solutions were stored on ice while performing the experiments.

Checkerboard microdilution assays were conducted to determine the MIC of individual agents (antimicrobial or enzyme) and their combinations against preformed biofilms in polystyrene 96-well microtiter plates. To distinguish the MIC measured against preformed biofilms from the one previously determined against planktonic cells (MIC-Plank), the former will be designated as MIC-Bio. MIC-Bio was quantified using the crystal violet assay, and absorbance was measured at OD_590nm_. Briefly, a 24 - hour old *B. cepacia* culture was adjusted to approximately 10^4^ CFU/mL in mYDC broth, and 200 µL of this suspension was added to each well of a polystyrene 96-well microtiter plate in a 6 × 7 matrix format. Plates were incubated at 37 °C for 48 hours to allow biofilm formation. After the incubation, the planktonic cells in each well were gently removed and washed twice with sterile saline solution (0.85%) to yield pre-formed biofilms for assessing the efficacy of enzyme-antimicrobial combinations. In a separate sterile 96-well plate, selected antimicrobials and enzymes were prepared at different concentrations, resulting in 6 combinations (**Table 2**). The prepared combinations were then added to the appropriate wells containing the pre-formed biofilms, and plates were incubated at 37°C for an additional 24 hours. After the incubation, the planktonic cells in each well were gently removed, and the wells were washed three times with sterile saline solution (0.85%). The excess water was allowed to dry by holding the plates at 55°C for 20 minutes. Biofilm formation was quantified by crystal violet assay using 250 µL of 0.1% crystal violet (Becton, Dickinson and Company) as described previously. The fractional inhibitory concentration index (FICI) for enzyme-antimicrobial combinations was calculated, as described previously (Liu et al., 2021), using the following equation:

**Table 2:** Concentrations of antimicrobial and enzyme combinations for testing their synergy against preformed biofilm in the checkerboard assay.

Combination	Concentration range
Ciprofloxacin α-amylase	1.0 - 31 μg/mL
	156.2–2500 μg/mL
Meropenem α-amylase	1.0 - 63 μg/mL
	156.2–2500 μg/mL
Ciprofloxacin Proteinase K	1.0 - 31 μg/mL
	31.25–500 μg/mL
Meropenem Proteinase K	1.0 - 63 μg/mL
	31.25–500 μg/mL
Meropenem DNase I	1.0 - 63 μg/mL
	0.00625 U/µL–0.01 U/µL
Ciprofloxacin DNase I	1.0 - 31 μg/mL
	0.00625 U/µL–0.01 U/µL


FICI=FIC(A)+FIC(B)

$$=\frac{MIC\;of\;antimicrobial\;A\;in\;combination}{MIC\;of\;antimicrobial\;A\;alone}\;\;+\frac{MIC\;of\;antimicrobial\;B\;in\;combination}{MIC\;of\;antimicrobial\;B\;alone}$$

The interaction was defined as follows: synergy, FICI ≤ 0.5; additivity, FICI > 0.5 to 1.0; and antagonism, FICI ≥ 2 (Fratini et al., 2017).

### 2.6 Testing biofilms on stainless-steel coupons

#### 2.6.1 Biofilm development

Biofilms were formed on the stainless-steel coupons (12.7 mm diameter, 304 stainless-steel disc coupons: Fisher Scientific). Briefly, sterile stainless-steel coupons were aseptically transferred to 24-well clear-bottom microtiter plates (Corning Costar; Fisher Scientific). The 24-hour-old culture of *B. cepacia* was diluted to ~10^4^ CFU/mL using mYDC broth, of which 1 mL was transferred to each well. The plates were incubated in the aerobic static and shaking (140 rpm) conditions at 37˚C up to 72 hours. Biofilm formed on the stainless-steel coupons was quantified by determining the total biofilm cell count (log_10_ CFU/coupon) at various time points (1, 12, 24, 48, and 72 hours) as follows. After the incubation, planktonic cells were carefully removed, and the stainless-steel coupons were washed three times with sterile saline (0.85%). The coupons were aseptically transferred into 50 mL Falcon tubes containing 5 mL of sterile saline and vortexed at high speed to dislodge the biofilm into the saline solution. To count the biofilm cells, 100 µL of the undiluted biofilm suspension tube was transferred to a microcentrifuge tube (Eppendorf, Enfield, CT, USA) containing 900 µL of mYDC broth, and serially diluted. The dilutions were spread-plated on mYDC agar, and colony counts were determined. This experiment was completed in triplicates.

#### 2.6.2 Synergistic combinations against pre-formed stainless-steel coupon biofilms

Biofilms were grown on stainless-steel coupons as previously described. After 48 hours of incubation for biofilm formation, the planktonic cells were carefully removed, and the coupons were washed three times with sterile saline (0.85%). For treatment, two experimental conditions were tested: (i) coupons were treated with 1 mL of individual applications of antimicrobials or enzymes, and (ii) coupons were treated with an enzyme-antimicrobial combination, with 0.5 mL of each component at their synergistic concentration as determined by the previously described checkerboard assay. Sterile media with coupons served as a negative control. The plates were incubated for an additional 24 hours at 37°C. To quantify changes in the biofilm mass on the coupons following treatments, the biofilm populations on the coupons were determined as described previously.

#### 2.6.3 Evaluation of the biofilm by scanning electron microscope

Scanning electron microscopy (SEM) was used to examine *B. cepacia* biofilms on stainless-steel coupons, according to a previously reported method (Tirpanci Sivri et al., 2023). Biofilm was formed on stainless-steel coupons, as described in a previous section, and each coupon was incubated for 24 hours with the following enzymes and antimicrobials at concentrations of their synergistic or additive effects: (i) α-amylase (625 μg/mL) and ciprofloxacin (4 μg/mL) individually and in combination, and (ii) α-amylase (1250 μg/mL) and meropenem (4 μg/mL) individually and in combination, with 48-hour-old biofilm coupons serving as the untreated control. Following the incubation, each coupon was washed twice with phosphate buffered saline (PBS) and fixed for 24 hours at ~22°C with 2.5% glutaraldehyde solution (Sigma-Aldrich, Inc.), made of 25% glutaraldehyde diluted 1:10 in 0.1 M phosphate buffer. After incubation, coupons were washed twice with PBS, dehydrated using ethanol gradients (25%, 50%, 70%, 85%, and 95%) for 10 minutes each, followed by two 30-min of 100% ethanol treatments. The coupons were coated with 10 nm iridium, and SEM images were obtained using the Thermo Scientific Trinity Detection System, with a T2 detector operating at 5 kV.

### 2.7 Statistical analysis

Data were analyzed using a commercial statistical analysis software (GraphPad Prism 9.0.0; GraphPad software, San Diego, CA, USA). The analysis of variance (ANOVA) was paired with Tukey’s test to rank pairs for multiple comparisons. Tests with *p* < 0.05 were considered significant.

## 3 Results

### 3.1 Growth behavior of planktonic *Burkholderia cepacia*

**Figure 1:**
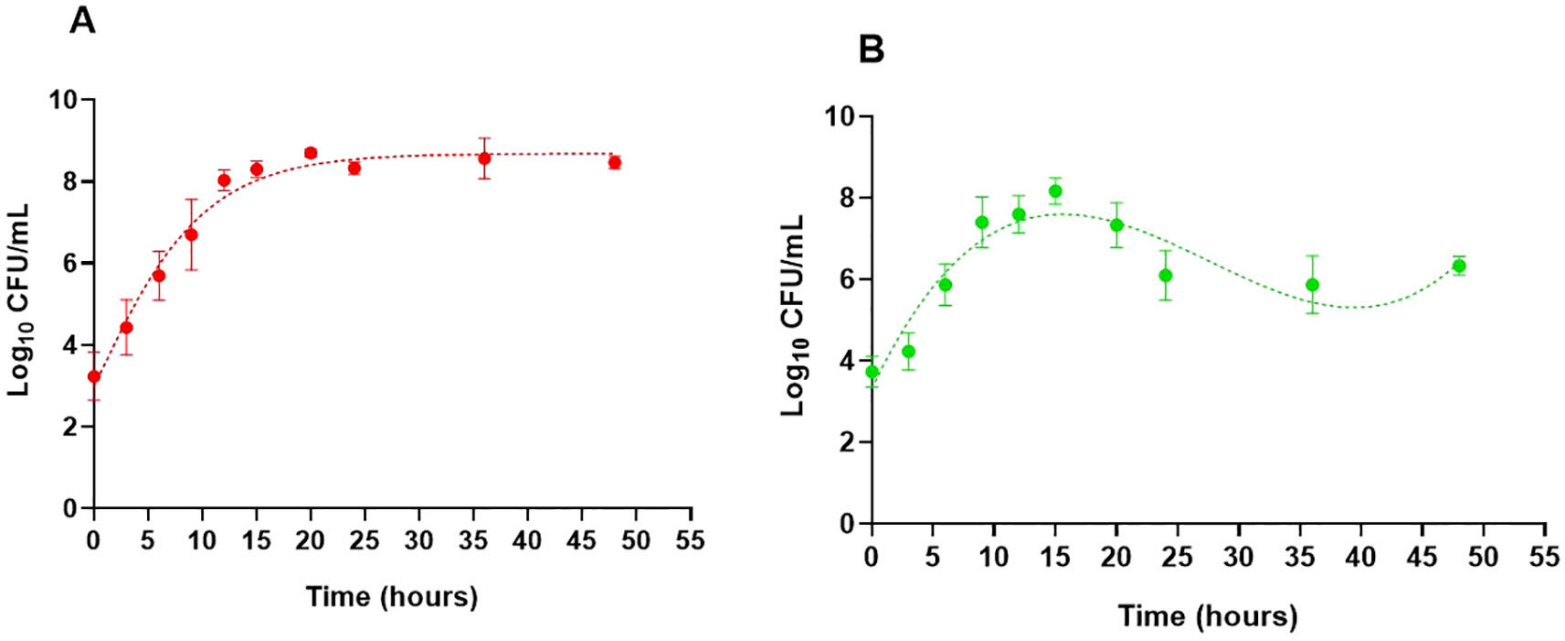
Growth behavior of *Burkholderia cepacia* ATCC 25416 in two nutritionally different media under static incubation at 37°C, measured as planktonic cell populations (log_10_ CFU/mL). Tryptone yeast extract dextrose broth **(A)** and modified yeast extract dextrose calcium carbonate broth **(B)**. Each data point represents the mean ± SD of three independent experiments. Dotted lines show growth curves predicted by the Gompertz model **(A)** and third-order polynomial **(B)**. For **(B)**, growth parameters were determined using the Gompertz model for the first 15 hours of incubation, during which both fits coincide.


The growth curves of *B. cepacia* in TYD and mYDC broths, incubated under static conditions at 37 °C, are illustrated in **Figure 1**. A typical growth curve was observed in the nutrient-rich, TYD broth, where the planktonic population steadily increased from an initial value of 3.2 ± 0.58 log_10_ CFU/mL at 0 hour to a maximum of 8.7 ± 0.08 log_10_ CFU/mL by the 20^th^ hour of incubation and remained steady throughout the remainder of the incubation period. The predicted growth, fitted using the Gompertz model, was governed by the following equation:

$$Y=3.02+5.57\;e^{-e}{-}^{0.24\;}{(}_{t-4.65)}$$

where *“Y”* is the *B. cepacia* population (log_10_ CFU/mL) and “*t”* is the incubation time (hour). Based on the model’s parameters, the estimated maximum growth rate was 0.24 log_10_ CFU/mL/hour, the predicted maximum growth was 8.6 log_10_ CFU/mL, and the correlation coefficient (R^2^) was 0.99, indicating a good fit of data by the model.

The growth behavior of *B. cepacia* in the mYDC broth was different than that observed in the TYD broth (**Figure 1**). Despite its poor nutritional composition, mYDC broth supported typical growth behavior during the first 15 hours of incubation, which was evident when this phase of growth was fitted using the Gompertz model (0.99 R2):


$$Y=3.68+4.51\;e^{-e-0.36\;\left(t-4.96\right)}$$

Growth parameters predicted by the model revealed a higher maximum growth rate (0.36 log_10_ CFU/mL/hour) and a lower maximum growth (8.2 log_10_ CFU/mL) in mYDC broth when compared with the growth patterns exhibited in TYD broth. After the growth reached its peak at 15^th^ hour (8.2 log_10_ CFU/mL, measured as well as model-predicted), the measured population decreased during the subsequent 21 hour to 5.9 log_10_ CFU/mL (2.3 log decrease), then increased again during the following 12 hours to 6.3 log_10_ CFU/mL (0.4 log increase). This cyclic growth pattern in the mYDC medium suggests a phenotypic switch from the planktonic state to biofilm-associated cells’ adherence to the surfaces of the polystyrene microtiter plate, which was followed by biofilm maturation and release of cells from the biofilm matrix. Expression of this cyclic behavior in mYDC broth but not in TYD broth is probably induced by the nutrient-limited conditions of the former medium.

### 3.2 Biofilm development as affected by growth media and incubation conditions

**Figure 2:**
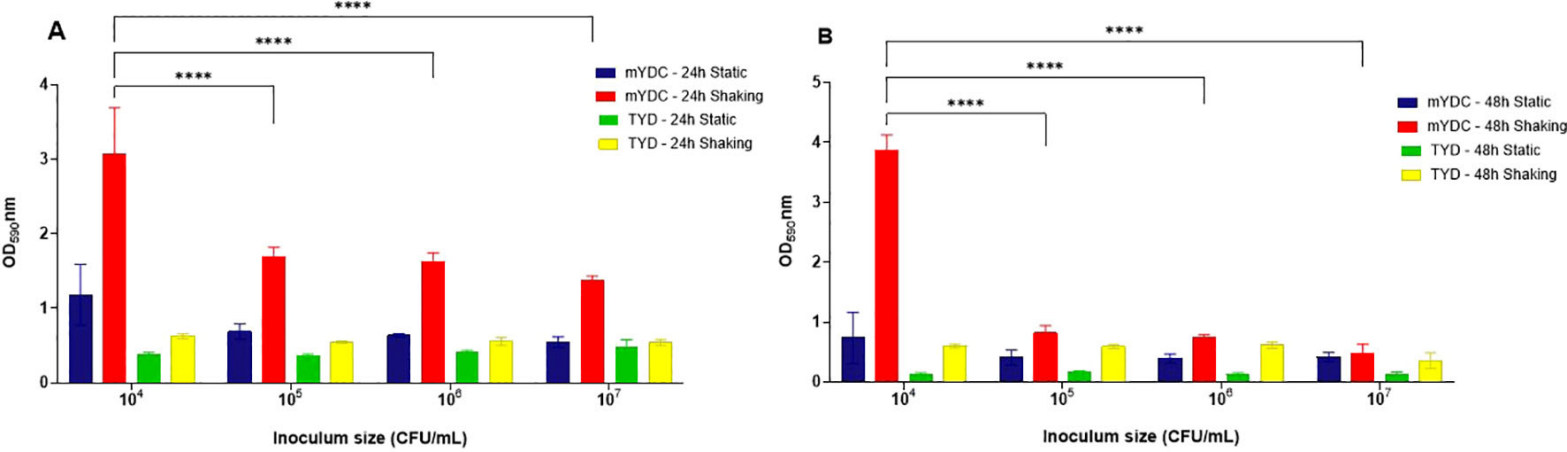
*Burkholderia cepacia* biofiilm development as affected by four inoculum sizes (10^4^–10^7^ CFU/mL), two microbiological broth media (tryptone yeast extract dextrose, TYD, and modified yeast extract dextrose calcium carbonate, mYDC), incubation times (24 and 48 hours) and aerobic incubation conditions (static and shaking). **(A, B)** represent biofilm growth observed at 24 and 48 hours, respectively. Results shown as mean ± standard deviation from four replicates. Two-way ANOVA was used to analyze the effect of inoculum size. (****), *p* < 0.0001.

When biofilm development was quantified using the colorimetric crystal violet assay (OD_590nm_) (**Figure 2**), the largest biofilm density was observed at an inoculum size of 10^4^ CFU/mL under shaking conditions (140 rpm) in mYDC broth, with optical densities (OD_590nm_) of 3.0 ± 0.62 after 24 hours and 3.8 ± 0.25 after 48 hours of incubation. The significant difference in biofilm density (*p* < 0.0001) was observed with the 10^4^ CFU/mL inoculum size compared to the other inocula (**Figure 2**). Compared to shaking conditions, biofilm density under aerobic static incubation was consistently smaller across all inoculum sizes (*p* < 0.0001), with the largest OD_590nm_ being 1.1 ± 0.41 for 10^4^ CFU/mL in mYDC broth. Compared to mYDC broth, biofilm formation in TYD broth was comparatively smaller across all incubation conditions and inoculum sizes, with OD_590nm_ values below ~1.0. These findings indicate that TYD broth, being nutrient-rich, likely promoted the planktonic growth rather than biofilm formation, which is often induced under nutrient-limiting conditions, as in the case of mYDC broth.

### 3.3 The MIC of antimicrobials against planktonic cells

The MIC values of the antimicrobials tested against planktonic cells of *B. cepacia* (MIC-Plank) were interpreted using the standard CLSI M100 Performance Standards for Antimicrobial Susceptibility Testing, 33^rd^ Edition, 2023 (Lewis et al., 2023). The MIC values ranged from 1000 μg/mL to 4 μg/mL for the tested antimicrobials (**Table 3**). The highest MIC (1000 µg/mL) was observed for erythromycin, whereas the lowest MICs, 4.0 and 8.0 μg/mL, were observed against ciprofloxacin and meropenem, respectively; for these two antibiotics, the absence of resazurin color change corresponded to the absence of turbidity at OD_600_ nm. According to the CLSI MIC breakpoints for the *B. cepacia* complex, the test bacterium was resistant to all tested antimicrobials except ciprofloxacin and meropenem, to which intermediate susceptibility was exhibited (**Table 3**).

**Table 3:** The minimum inhibitory concentration (MIC; µg/mL) values of different antimicrobials against *Burkholderia cepacia* planktonic cells (MIC-Plank).

Antimicrobial agents	Minimum inhibitory concentration (μg/mL)	MIC breakpoints per CLSI guidelines^a^ (μg/mL)			Interpretation
		S^b^	I^c^	R^d^	
Tetracycline	256	≤ 4	8	≥16	Resistant
Chloramphenicol	125	≤ 8	16	≥32	Resistant
Kanamycin sulfate	625	No CLSI breakpoint			–
Ceftazidime	62.5	≤ 8	16	≥32	Resistant
Erythromycin	1000	No CLSI breakpoint			–
Ampicillin	–	No CLSI breakpoint			Resistant
Ciprofloxacin	4.0	≤ 2	4	≥16	Intermediate
Meropenem trihydrate	8.0	≤ 4	8	≥16	Intermediate

^a^CLSI M100 Performance Standards for Antimicrobial Susceptibility Testing, 33^rd^ Edition, 2023.

^b^Sensitive; ^c^Intermediate; ^d^Resistant.

### 3.4 Enzyme-antimicrobial synergy against pre-formed biofilms

**Figure 3:**
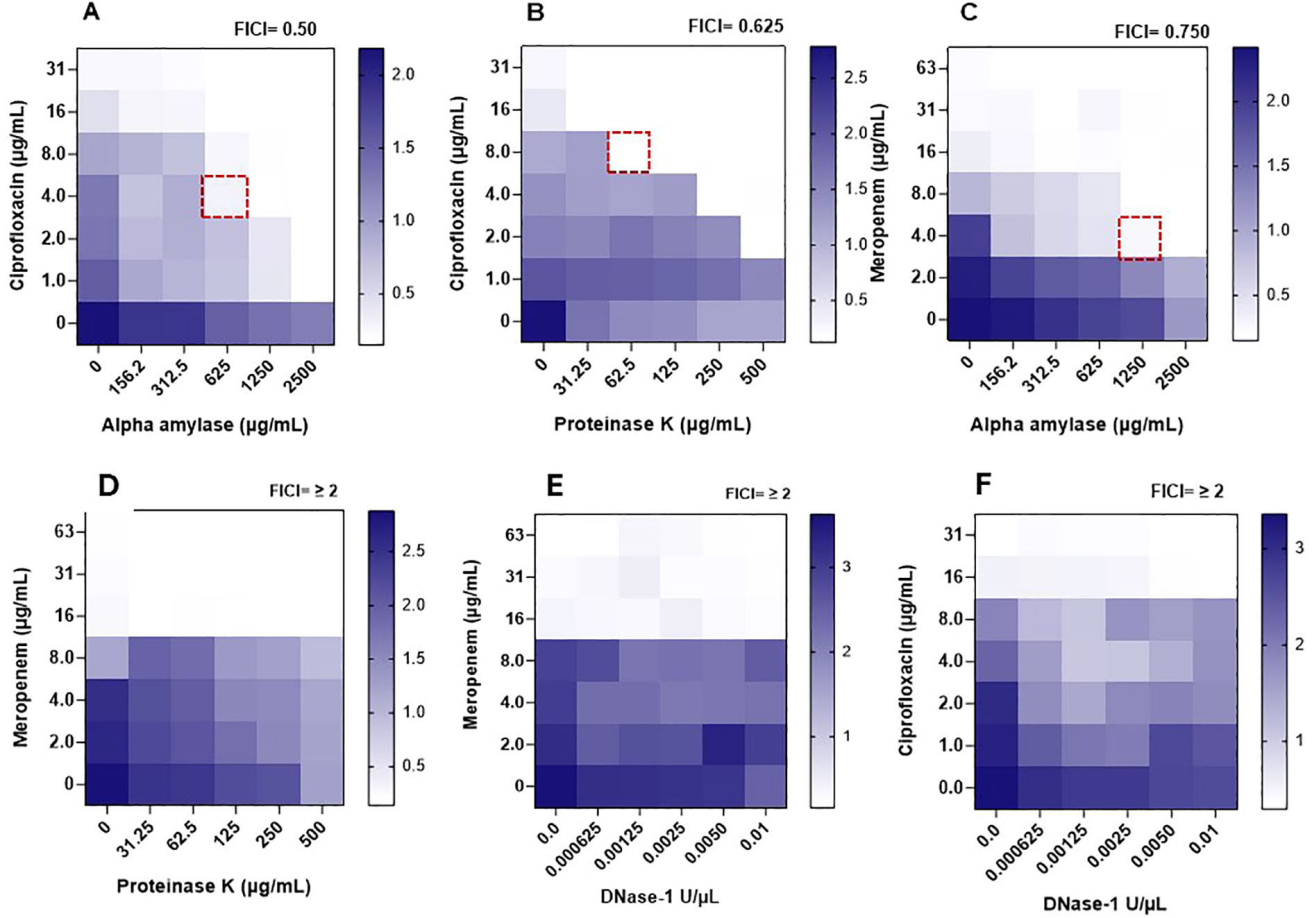
Heatmaps of optical densities (OD_590nm_) for crystal violet adhering to *Burkholderia cepacia* biofilms in assay wells during determining the synergistic activity between enzymes and antimicrobials against the preformed biofilm measured through the checkerboard assay. A dotted square represents the combination’s minimum inhibitory concentration against preformed biofilm (MIC-Bio). **(A)** α-amylase/ciprofloxacin. **(B)** Ciprofloxacin/proteinase K. **(C)** α-amylase/meropenem. **(D)** Meropenem/proteinase K. **(E)** Meropenem/DNase I. **(F)** Ciprofloxacin/DNase I. Estimated enzymes’ anti-biofilm MIC (MIC-Bio) were: α-amylase, >2500 µg/mL; proteinase K, > 500 µg/mL; DNase 1, > 0.01 U/µL. A scale bar, to the right of a heatmap, indicates OD_590nm_ readings of different intensities of crystal violet colors, with dark blue regions indicating areas of high cell density.

The MICs of selected antimicrobials were tested, alone or in combination with potentially synergistic enzymes, against preformed *B. cepacia* biofilms (MIC-Bio), and the results are shown in **Figure 3** and analyzed in **Table 4**. When applied individually, ciprofloxacin at four times its MIC-Plank was required to eliminate *B. cepacia* preformed biofilm, whereas meropenem at twice its MIC-Plank eliminated the preformed biofilm (**Tables 3**, **4**). However, the two antimicrobials eliminated the biofilm at levels lower than their individual MIC-Bio when combined with the matrix-degrading enzymes. Out of the six combinations (**Table 4**), α-amylase and ciprofloxacin possessed the highest synergistic effect (FICI, 0.50), whereas proteinase K and ciprofloxacin (FICI, 0.625), and α-amylase and meropenem (FICI, 0.750) displayed an additive effect (**Table 4**). In contrast, DNase I in combination with either ciprofloxacin or meropenem exhibited an antagonistic effect (FICI, ≥ 2), showing no impact on biofilm eradication. Additionally, the combination of meropenem with proteinase K (FICI≥ 2) displayed an antagonistic interaction.

**Table 4:** The minimum inhibitory concentration of selected antimicrobials, synergetic enzymes, and their combinations, against *Burkholderia cepacia* preformed biofilms (MIC-Bio) as determined by the checkerboard assay and assessing the mode of enzyme-antimicrobial interaction using the fractional inhibitory concentration (FIC) of each agent and the FIC index of the combinations.

Antimicrobial combinations	MIC-Bio Individual^a^	MIC-Bio in combination^a^	FIC	FICI	Interaction
Ciprofloxacin α-amylase	16 μg/mL	4 μg/mL	0.25	0.50	Synergistic
	>2500 μg/mL^b^	625 μg/mL	0.25		
Ciprofloxacin Proteinase k	16 μg/mL	8 μg/mL	0.5	0.625	Additive
	>500 μg/mL^b^	62.5 μg/mL	0.125		
Meropenem α-amylase	16 μg/mL	4 μg/mL	0.25	0.750	Additive
	>2500 μg/mL^b^	1250 μg/mL	0.50		
Meropenem Proteinase K	16 μg/mL	16 µg/mL	1	≥ 2	No effect
	>500 μg/mL^b^	>500 µg/mL	1		
Meropenem DNase I	16 μg/mL	16 µg/mL	1	≥ 2	No effect
	>0.01 U/µL^b^	>0.01 U/µL	1		
Ciprofloxacin DNase I	16 µg/mL	16 µg/mL	1	≥ 2	No effect
	>0.01 U/µL^b^	>0.01 U/µL	1		

^a^Deduced from **Figure 3**.

^b^All tested concentrations of the enzymes did not inhibit biofilm formation, hence their MICs were set at the highest concentration tested and thus considered estimates.

### 3.5 Evaluation of *Burkholderia cepacia* adhesion on stainless-steel coupons under shaking and static incubation

**Figure 4:**
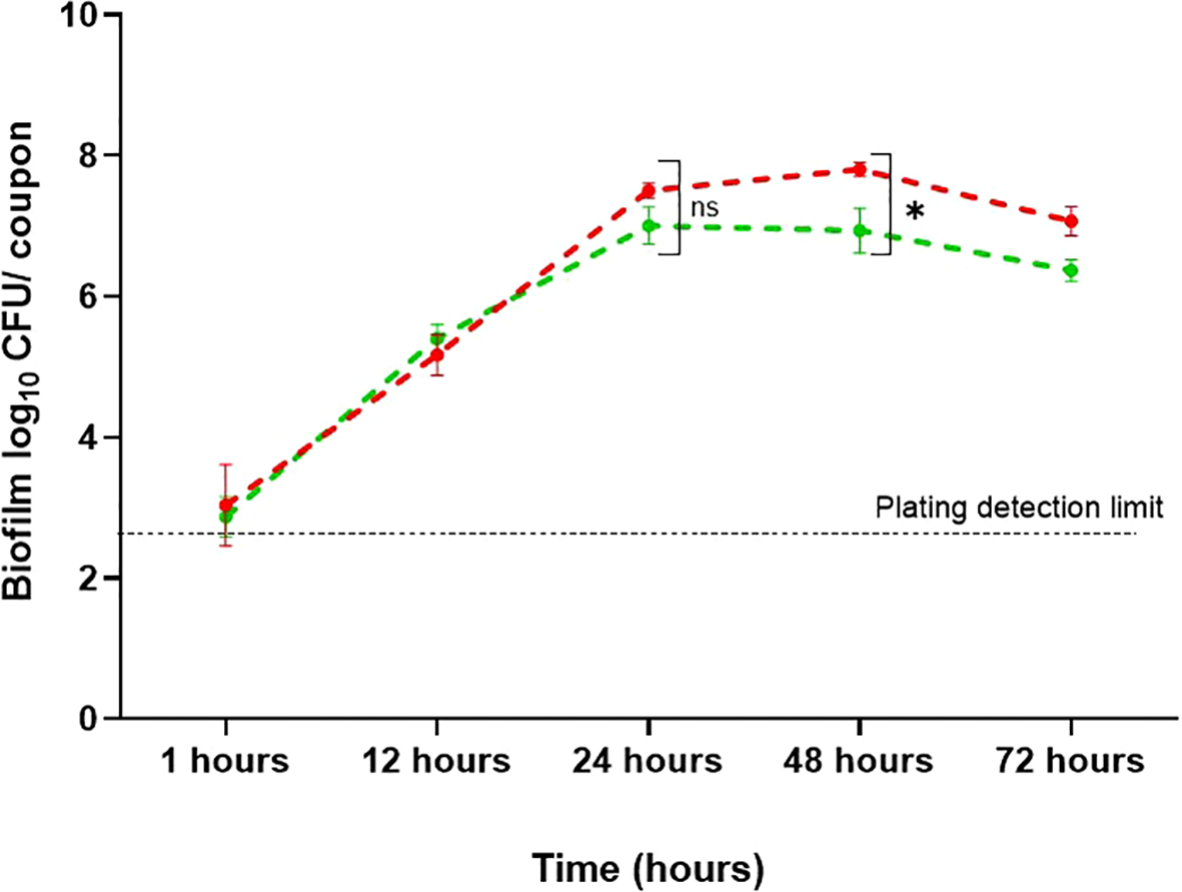
*Burkholderia cepacia* biofilm development on stainless-steel surfaces under aerobic shaking and static condition for 72 hours at 37°C. Each data point represents mean ± standard deviation from three replicates. Red line, static incubation; green line, shaking condition. Asterisk denotes significant difference (*p* < 0.01), whereas ns denotes non-significant difference (*p* > 0.05).

Stainless-steel is a widely used food contact material in processing equipment due to its corrosion resistance, ease of cleaning, and exceptional mechanical strength (Ciolacu et al., 2022). To mimic an industrial environment, biofilms were developed on stainless-steel coupons, and conditions that affect the adhesion of *B. cepacia* to their surfaces were investigated. Considering the suitability of mYDC medium and 10^4^ CFU/mL inoculum for robust biofilm development in the wells of microtiter plates, these conditions were applied in stainless-steel experiments. During the first 24 hours of incubation (**Figure 4**), the biofilm populations reached 7.5 ± 0.1 and 7.0 ± 0.26 log_10_ CFU/coupon, under aerobic static and shaking conditions, respectively, but the difference between these two populations was not significant (*p* > 0.05). During the subsequent 24 hours of incubation, the biofilm populations did not increase considerably, but the maximum growth of 7.8 ± 0.10 and 6.9 ± 0.32 log_10_ CFU/coupon was reached for biofilms formed under aerobic static and shaking incubation, respectively, and these two populations were significantly different (*p* < 0.01). During the last 24 hours of the 72-hours incubation, biofilm log_10_ CFU/coupon decreased slightly, possibly due to nutrient depletion or biofilm detachment.

### 3.6 Synergistic antimicrobial combinations against preformed biofilm on stainless-steel coupons

**Figure 5:**
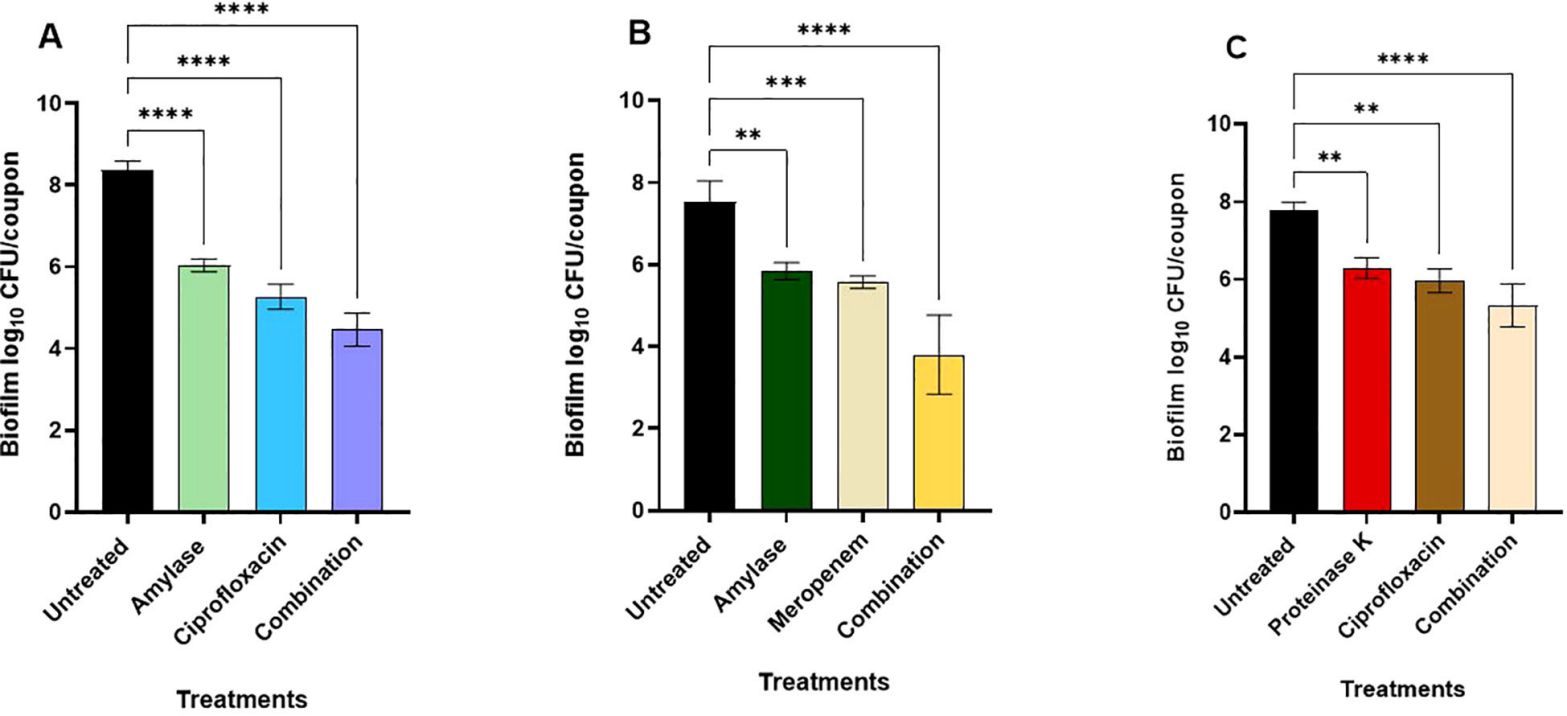
Effects of enzyme-antimicrobial combinations on preformed biofilms of *Burkholderia cepacia* on stainless-steel coupons during incubation in mYDC medium for 24 hours at 37°C. **(A)** α-amylase (625 μg/mL) and ciprofloxacin (4 μg/mL). **(B)** α-amylase (1250 μg/mL) and meropenem (4 μg/mL). **(C)** Proteinase K (62.5 μg/mL) and ciprofloxacin (8 μg/mL). Enzyme-antimicrobial combinations compared to individual applications, *p* < 0.0001. Error bars, ± standard deviation from three independent experiments. (****), *p* < 0.0001; (***), *p* < 0.001; **), *p* < 0.01.

The most promising enzyme-antimicrobial combinations, based on the checkerboard assay in the microtiter plates (**Table 4**), were tested against *B. cepacia* biofilm that was pre-formed on the stainless-steel coupons. Overall, significant biofilm reduction was observed in all treatment groups compared to the untreated control. One of these combinations was α-amylase and ciprofloxacin at 625 μg/mL and at 4 μg/mL, respectively (**Figure 5A**). The *B. cepacia* biofilm on stainless-steel coupons significantly decreased (*p* < 0.0001) from 8.4 ± 0.2 log_10_ CFU/coupon to 6.03 ± 0.2, 5.3 ± 0.3, and 4.5 ± 0.4 log_10_ CFU/coupon with α-amylase, ciprofloxacin, and a combination thereof, respectively (**Figure 5A**). In the combination of α-amylase (1250 μg/mL) and meropenem (4 μg/mL), *B. cepacia* biofilm on stainless-steel coupons significantly decreased (*p* < 0.0001) from 7.5 ± 0.5 log_10_ CFU/coupon to 5.8 ± 0.2, 5.6 ± 0.2, and 3.8 ± 1.0 log_10_ CFU/coupon with α-amylase, meropenem, and a combination of both agents, respectively (**Figure 5B**). Hence, the last combination significantly (*p* < 0.0001) degraded the pre-existing biofilm as compared to the untreated control. When proteinase K (62.5 μg/mL) and ciprofloxacin (8 μg/mL) were tested, the count of the preformed biofilm decreased significantly (*p* < 0.0001), from 7.7 ± 0.2 log_10_ CFU/coupon to 6.0 ± 0.3, 6.3 ± 0.3, and 5.3 ± 0.6 log_10_ CFU/coupon with ciprofloxacin, proteinase K, and their combination, respectively (**Figure 5C**). Overall, α-amylase in combination with either meropenem or ciprofloxacin demonstrated greater efficacy in reducing preformed *B. cepacia* biofilms, compared to combinations with proteinase K.

### 3.7 Scanning electron microscopy reveals the structure of biofilms

**Figure 6:**
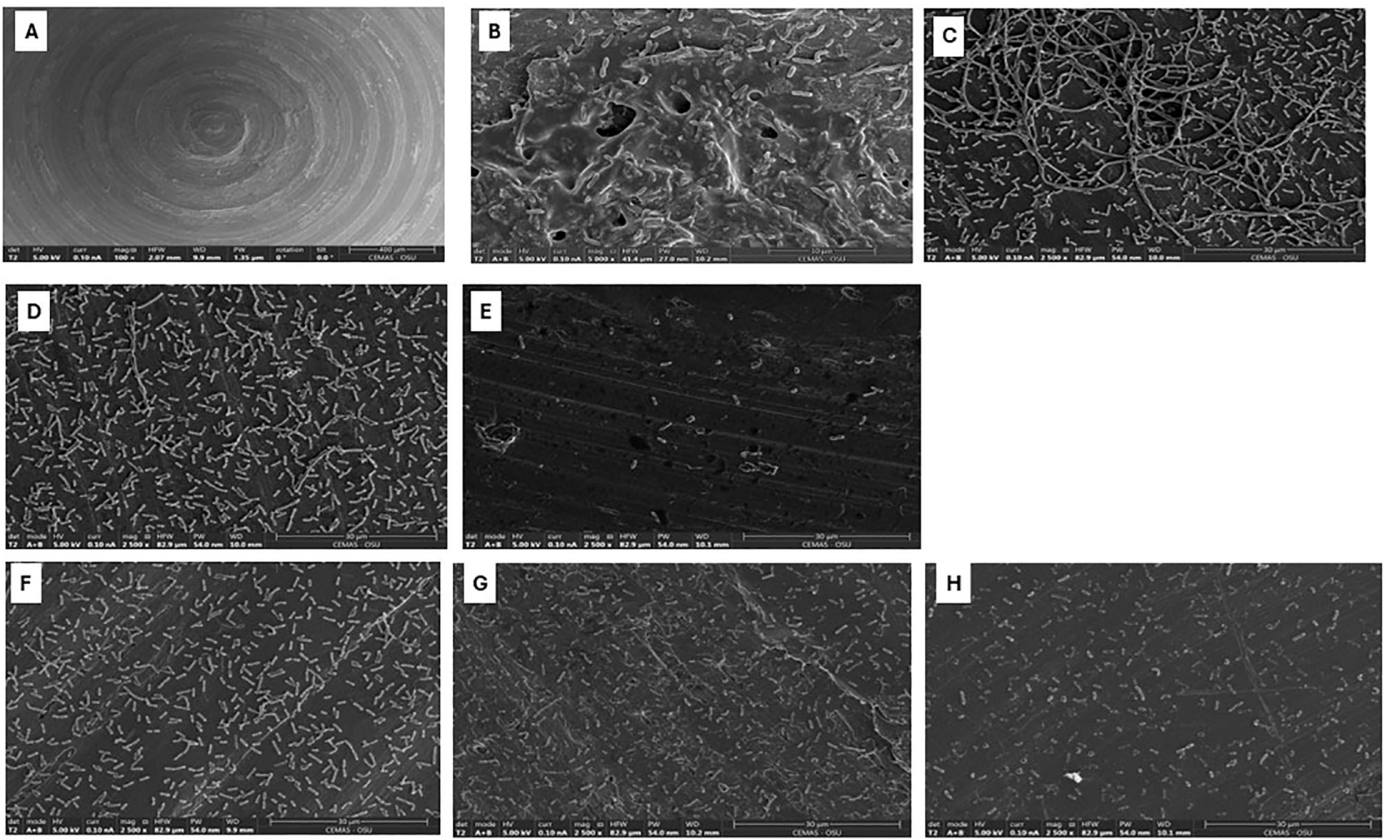
Scanning electron micrographs of *Burkholderia cepacia* biofilms on stainless-steel coupons subjected to selected concentrations of ciprofloxacin, meropenem, or α-amylase-antimicrobial combinations. **(A)** Surface of unused stainless-steel coupon (bar = 400 µm). **(B)** Untreated 48 hours biofilm (bar = 10 µm). **(C)** Treated with ciprofloxacin, 4 µg/mL (bar = 30 µm). **(D)** Treated with α-amylase, 625 µg/mL (bar = 30 µm). **(E)** Treated with α-amylase and ciprofloxacin combination (bar = 30 µm). **(F)** Treated with meropenem, 4 µg/mL (bar = 30 µm). **(G)** Treated with α-amylase, 1250 µg/mL (bar = 30 µm). **(H)** Treated with α-amylase and meropenem combination (bar = 30 µm).

The scanning electron microscope was used to assess the morphological changes of *B. cepacia* biofilms formed on stainless-steel surfaces, in response to promising treatments deduced from the checkerboard assay (**Table 4**). Uninoculated stainless-steel coupons (**Figure 6A**) had a generally smooth surface with minor cracks and irregularities, showing the baseline topography before bacterial colonization. In untreated control samples (**Figure 6B**), *B. cepacia* established dense, well-developed biofilms. Cells were embedded in a thick EPS matrix that firmly adhered to the substrate. Morphologically, cells within the EPS appeared as short rods, whereas those cells outside the matrix were longer rods. Bacterial morphology changed significantly after treatment with ciprofloxacin alone (**Figure 6C**). The cells were elongated and filamentous, indicating impaired septation and potential interference with DNA replication and cell division (Wickens et al., 2000). Biofilm samples treated with α-amylase alone (625 μg/mL; **Figure 6D**) showed a noticeable reduction in biofilm biomass and EPS matrix. The cells appeared dispersed and loosely attached to the surface, lacking the cohesive structure typical of mature biofilms. The combination of α-amylase and ciprofloxacin (**Figure 6E**) produced a synergistic effect consistent with the previous results (**Figure 3**). Even though individual cells were present, cell density was significantly reduced, and there was no EPS or structured biofilm. The synergism between the enzymatic degradation of EPS and increased antimicrobial penetration probably contributed to the improved antimicrobial efficacy. The second combination to which biofilm was exposed was α-amylase (1250 μg/mL) and meropenem (4 μg/mL) (**Table 4**; **Figure 6F**). Meropenem treatment caused morphological changes in cells, such as V-shaped cell arrangements. Exposure to the α-amylase alone had the same effect as previously mentioned; there was no EPS production or thick biofilm mass (**Figure 6G**). However, samples treated with α-amylase and meropenem together showed visible morphological changes (**Figure 6H**). The bacterial population seemed to have clearly decreased, and the remaining cells showed deformed shapes, including shrinking and spheroplast-like appearances.

## 4 Discussion

*B. cepacia* is an underexplored opportunistic pathogen and a food spoilage microorganism. The biofilm-forming capabilities of the bacterium make it an ideal model for investigating a potential foodborne hazard. The present study evaluated the ability of the *B. cepacia* ATCC 25416 strain to form biofilm on polystyrene (microtiter plate surface) and stainless-steel coupons. Antimicrobials combined with enzymes such as DNase I that degrades extracellular DNA, α-amylase that breaks down extracellular polysaccharides, and proteinase K that hydrolyzes proteins (Al-Madboly et al., 2024) were tested against preformed *B. cepacia* biofilms as a potential alternative to traditional biofilm control approaches.

When *B. cepacia* was incubated in two different microbiological broths (**Table 1**, **Figure 1**), it exhibited a typical growth pattern in the nutrient-rich TYD medium and a non-typical pattern in the nutrient-deficient mYDC medium. These findings suggest that TYD broth supported planktonic cell multiplication and produced a familiar sigmoid growth curve, whereas mYDC broth gave a cyclic growth behavior. After *B. cepacia* population reached the stationary phase in mYDC broth, it decreased by 2.3 log_10_ CFU/mL, then partially recovered by the end of the incubation period. To explain this cyclic growth phenotype, it is conceivable that nutrient deficiency in mYDC broth encouraged the early phenotypic switch from a planktonic to a biofilm state, and after the surface biofilm matured, cell dispersion to a planktonic state started. Exposure to stress is known to induce a transition from the planktonic state to the biofilm mode of growth, prompting bacteria to form biofilms as a survival and resource optimization strategy (Rumbaugh and Sauer, 2020; Samrot et al., 2021). Researchers also reported the dispersal of the sessile biofilm cells while transitioning to the planktonic mode of growth (Rumbaugh and Sauer, 2020). In a previous study, researchers found that nutrient depletion in bacterial biofilms can trigger the production of the EPS matrix (Zhang et al., 2014).

The influence of incubation conditions, inoculum size, and media type on *B. cepacia* biofilm was optimized (**Figure 2**). The smallest inoculum size we tested (10^4^ CFU/mL) produced significantly (*p* < 0.0001) more biofilm biomass as compared to larger inocula. It is likely that while the initial small population multiplies, cells have greater opportunities to differentiate into the biofilm state, but this assumption needs further investigation. Other researchers suggested that large cell inocula have high competition for space and substrate, and these cells progress into the stationary phase earlier than those of lower inoculum levels (Lichtenberg et al., 2022). In microtiter plate wells, low-speed shaking conditions promoted biofilm formation more effectively than static conditions (**Figure 2**). Other researchers (Jara et al., 2021) found that shaking incubation resulted in biofilms on glass coverslips that were denser (~8 log_10_ CFU/cm²) than those of static incubation (~7 log_10_ CFU/cm²).

Determining the MIC of 8 antimicrobials against planktonic *B. cepacia* showed that ciprofloxacin and meropenem exhibited the lowest MIC values (4.0 and 8.0 μg/mL, respectively; **Table 3**). High concentrations of other antimicrobials, such as tetracycline (125 μg/mL), did not have any inhibitory effect against the cells of *B. cepacia*, as this bacterium is known for its intrinsic resistance mechanisms, including efflux pumps and biofilm-mediated protection (Rhodes and Schweizer, 2016). For instance, *B. cenocepacia* has at least six efflux pumps of the RND family that are implicated in drug resistance (Rhodes and Schweizer, 2016). *B. cepacia* complex exhibits intrinsic resistance to multiple antimicrobial classes, including penicillin’s cephalosporins (except ceftazidime), monobactams, carbapenems (except meropenem), polymyxins, aminoglycosides, and fosfomycin (Demir et al., 2022).

Over the past decade, the application of enzymes has gained recognition as a promising strategy for combating biofilms on food industry surfaces (Abdelhamid and Yousef, 2023). Recently, the potential use of EPS-degrading enzymes as a strategy for biofilm control was investigated (Lim et al., 2019). In a previous study, complete removal of biofilm cells was achieved when alkaline α-amylase and protease were combined with peracetic acid treatment. In contrast, using enzymes alone resulted in only 18% biofilm cell removal (Iñiguez-Moreno et al., 2021). The enzymes likely degraded the biofilm matrix, enhancing the penetration of peracetic acid and allowing it to effectively inactivate the cells embedded within the biofilm. In the current study, we evaluated the effectiveness of α-amylase, DNase I, and proteinase K in disrupting pre-formed *B. cepacia* biofilms (**Figure 3**). The checkerboard assay demonstrated the potential of the enzyme in enhancing antimicrobial efficacy. At sub-MIC-Bio concentrations (**Table 4**), ciprofloxacin exhibited the highest synergistic effect when combined with α-amylase, whereas α-amylase with meropenem and ciprofloxacin with proteinase K showed additive effect. Glycoside hydrolases, such as α-amylase, dispersin B, and alginate lyase, can hydrolyze polysaccharide components and weaken the biofilm matrix, thereby assisting in eliminating biofilms (Efremenko et al., 2023). Amylases are a prominent group of enzymes used in cleaning processes, with α-amylase and glucoside amylase being the most studied types. Together, they account for approximately 25% of the global enzyme market (Sundarram and Murthy, 2014). Thus, α-amylase mediates the hydrolysis of the polysaccharide’s α-1,4-glycosidic bonds, forming low-molecular-weight molecules in the process (Lahiri et al., 2021). It is plausible that α-amylase allowed ciprofloxacin to penetrate through the polymeric matrix and thereby adversely affecting the cells by blocking the DNA gyrase (Serizawa et al., 2010). DNA gyrase is made up of subunits A and B, and ciprofloxacin is thought to inhibit subunit A, leading to exonucleolytic degradation and cell damage (Shariati et al., 2022). When we combined α-amylase with meropenem, the enzyme likely improved the access of the antimicrobial to the penicillin-binding proteins located on the cytoplasmic membrane, leading to cell lysis and disruption of the biofilm architecture (Wickremasinghe et al., 2021).

In the current study, Proteinase K in combination with ciprofloxacin reduced *B. cepacia* preformed biofilm. Proteinase K resembles naturally produced proteases and may be used to facilitate biofilm disruption by breaking surface proteins (Eladawy et al., 2020). It breaks down peptide bonds that are close to the carboxylic groups of aromatic and aliphatic amino acids (Kumar Shukla and Rao, 2013). In this study, when the meropenem and proteinase K combination was tested, meropenem at its MIC-Bio seemed to decrease the biofilm, but there was no synergistic effect between these two agents. Using DNase I, in combination with ciprofloxacin or meropenem, did not result in synergistic effects. Extracellular DNA is known to play an important structural role as a component of various bacterial biofilms and to protect bacterial cells from environmental stresses (Lim et al., 2021). The lack of DNase activity in reducing the preexisting biofilm may be due to the low concentrations of DNase I we used in the current study or due to the EPS structure, which was not susceptible to DNase (Lim et al., 2021).

Food processing equipment is often made of stainless steel due to its strength, corrosion resistance, and durability. Type 304 stainless steel is one of the most versatile and widely used grades in the food industry (Pathirajah et al., 2022); it was therefore used in this study. Biofilm formation on stainless-steel coupons was better under static conditions at 48 h compared to mild shaking conditions (**Figure 4**). This observation is contrary to what was observed in the case of biofilm formation on the walls of 96-well polystyrene microtiter plates, where shaking conditions gave better results (**Figure 2**). This discrepancy could be attributed to the surface topography or the effect of shear stress caused by the slight movement of the coupons within the wells, which may have disrupted the biofilm and made it less stable.

As mentioned earlier, the use of enzyme-antimicrobial combinations is a good strategy to target preformed biofilms. Ciprofloxacin at minimal concentrations caused a significant decrease in biofilm cells formed on stainless-steel coupons (**Figures 5**, **6**). According to Schmitz et al. (2002), fluoroquinolones (e.g., ciprofloxacin), at high concentrations, exhibit bactericidal effect, but at lower concentrations, they exhibit bacteriostatic characteristics. In the current study, combining α-amylase with ciprofloxacin reduced biofilm population from 8.4 ± 0.2 to 4.5 ± 0.4 log_10_ CFU/coupon, representing a significant (*p* < 0.0001) reduction in biofilm mass. When combined with α-amylase that breaks down the EPS, ciprofloxacin penetrated the biofilm more deeply, and the previously protected biofilm cells became exposed to the antimicrobial agent. Meropenem at a low concentration significantly decreased the biofilm when compared to the untreated control. Combination of α-amylase with meropenem significantly (*p* < 0.0001) reduced the biofilm on stainless steel coupons from 7.5 ± 0.5 log_10_ CFU/coupon to 3.8 ± 1.0 log_10_ CFU/coupon, highlighting the effect of this combination against *B. cepacia* biofilms.

In the SEM analysis, the morphological changes observed in the biofilm upon treatment with ciprofloxacin were mainly filamentation and elongation of cells (**Figure 6C**). In contrast, α-amylase treatment alone did not induce cellular morphological changes; however, it effectively disrupted the biofilm matrix, with a noticeable absence of EPS and dense biofilm structures (**Figure 6D**). The combination of α-amylase and ciprofloxacin exhibited a significant reduction in the density of the attached cell and complete absence of biofilm EPS, highlighting a potential synergistic effect (**Figure 6E**). These observations are consistent with published reports that ciprofloxacin, like other DNA-damaging agents, triggers an SOS response, which controls DNA repair and cell filamentation. Filaments are a metabolically active phenotype that persists after antimicrobial removal (Shariati et al., 2022). Similar findings were reported by (Jesmina et al., 2023), who observed pronounced filamentation in *Escherichia coli* treated with ciprofloxacin, with even greater elongation occurring when used in combination with other agents. Overall, α-amylase caused the breakdown of the EPS, and the antibiotic caused morphological changes in the biofilm cells, which may provide a possible mechanism of the synergism between the two agents.

When *B. cepacia* biofilms were treated with α-amylase and meropenem combination, spherical/rounded spheroplasts with detached cells were visible (**Figure 6H**). Meropenem is a carbapenem, a class of β-lactam (Yang et al., 2023). β-lactams primarily target penicillin-binding proteins (PBPs), particularly PBP-1, -2, and -3, in Gram-negative bacteria. These proteins play a crucial role in the final stages of bacterial peptidoglycan assembly (Glen and Lamont, 2021). Inhibiting PBP-1 leads to rapid killing and lysis, while inhibiting PBP-2 and PBP-3 results in spherical, non-growing cells, or long filaments (Lang et al., 2021).

Based on the findings of the current study, combining enzymatic treatments with antimicrobials is a promising strategy for addressing biofilm-associated challenges caused by *B. cepacia*. This approach could be beneficial to the food industry if the clinically-relevant antimicrobial agents are replaced with approved food-grade antimicrobials. Combining enzymes with natural antimicrobials, such as bacteriocins, organic acids, or essential oils, could assist in biofilm control in a manner similar to what was observed in the current study. Supporting this idea, a recent work (Cervantes-Huamán et al., 2025) demonstrated that multi-enzyme blends (protease, amylase, and mananase) were synergistic with *Cinnamomum cassia* essential oil in disrupting mature *Listeria monocytogenes* and *Salmonella enterica* biofilms on stainless steel surfaces. Similarly, (Li et al., 2018) showed that the natural lipopeptide, paenibacterin, inhibited biofilm formation at very low (≤10 µg/mL) concentrations, disrupted preformed biofilms of *Listeria monocytogenes* under certain conditions, and down-regulated key biofilm-related genes (*prfA, agrA, flaA, fliG, flgE*), offering a gene-level mechanism of action.

Despite these advancements, implementing enzyme-based techniques in food processing presents significant challenges. The stability, cost, and usefulness of enzymes in industrial settings must be carefully considered. Temperature fluctuations, pH changes, or detergent/sanitizer exposure can impact enzyme activity. Furthermore, large-scale manufacturing and purification of enzymes could be costly, limiting their economic applicability. However, practical use of the approach presented in the current research requires additional optimization studies and cost-effectiveness analysis.

## 5 Conclusion

The current study addresses a critical knowledge gap by investigating parameters affecting biofilm formation of *B. cepacia*, a rarely explored opportunistic pathogen and a spoilage microorganism. *B. cepacia* formed a significant biofilm mass on a food contact surface (stainless steel). Biofilm matrix-degrading enzymes, in combination with conventional antimicrobials, destroyed *B. cepacia* preformed biofilm in a synergistic or additive manner. Combination of α-amylase and ciprofloxacin or meropenem, was effective in removing preformed biofilms, which were resilient when either the enzyme or the antimicrobial agent was used alone. The efficacy of α-amylase-antimicrobial combinations on stainless-steel surfaces highlighted their potential as a promising approach to combat biofilm-associated problems. The current study provides an essential proof-of-concept evidence, but alternative food-compatible enzyme-antimicrobial combinations need to be developed, and their efficacy optimized under pilot-scale conditions. Furthermore, factors such as safety validation, stability, cost-effectiveness, and regulatory compliance will need to be carefully addressed before these treatments can be integrated into industrial sanitation practices.

## Data Availability

The original contributions presented in the study are included in the article/supplementary material. Further inquiries can be directed to the corresponding authors.
